# Single-Molecule Real-Time Sequencing to Explore the Mycobiome Diversity in Malt

**DOI:** 10.1128/spectrum.00511-22

**Published:** 2022-09-26

**Authors:** Nan Long, Jinxin Liu, Jiali Liu, Ying Li, Yujiao Hou, Xiaofang Liao, Lidong Zhou, Linchun Shi, Weijun Kong

**Affiliations:** a Institute of Medicinal Plant Development, Chinese Academy of Medical Sciences and Peking Union Medical College, Beijing, China; b School of Traditional Chinese Medicine, Capital Medical University, Beijing, China; The National University of Singapore and the Genome Institute of Singapore

**Keywords:** fungal community, mycobiome diversity, single-molecule real-time sequencing, full-length ITS, toxigenic fungi, malt

## Abstract

This study determined the composition of fungal communities and characterized the enriched fungal species in raw and roasted malts via the third-generation PacBio-based full-length single-molecule real-time (SMRT) sequencing of the full-length amplicon of the internal transcribed spacer (ITS) region. In total, one kingdom, six phyla, 23 classes, 56 orders, 120 families, 188 genera, 333 species, and 780 operational taxonomic units (OTUs) were detected with satisfactory sequencing depth and sample size. Wickerhamomyces (56%), Cyberlindnera (15%), Dipodascus (12%), and Candida (6.1%) were characterized as the dominant genera in the raw malts, and Aspergillus (35%), Dipodascus (21%), Wickerhamomyces (11%), and Candida (3.5%) in the roasted malts. Aspergillus proliferans, Aspergillus penicillioides, and Wickerhamomyces anomalus represented the crucial biomarkers causing intergroup differences. Correlation analysis regarding environmental factors indicated that the water activity (a_w_) of the samples affected the composition of the fungal communities in the malts. In practice, special attention should be paid to the mycotoxin-producing fungi, as well as other fungal genera that are inversely correlated with their growth, to ensure the safe use of malt and its end products.

**IMPORTANCE** Fungal contamination and secondary metabolite accumulation in agricultural products represent a global food safety challenge. Although high-throughput sequencing (HTS) is beneficial for explaining fungal communities, it presents disadvantages, such as short reads, species-level resolution, and uncertain identification. This work represents the first attempt to characterize the fungal community diversity, with a particular focus on mycotoxin-producing fungi, in malt via the third-generation PacBio-based full-length SMRT sequencing of the ITS region, aiming to explore and compare the differences between the fungal communities of raw and roasted malts. The research is beneficial for developing effective biological control and conservation measures, including improving the roasting conditions, monitoring the environmental humidity and a_w_, and effectively eliminating and degrading fungi in the industry chain according to the diverse fungal communities determined, for the safe use of malts and their end products, such as beers. In addition, the third-generation SMRT sequencing technology allows highly efficient analysis of fungal community diversity in complex matrices, yielding fast, high-resolution long reads at the species level. It can be extended to different research fields, updating modern molecular methodology and bioinformatics databases.

## INTRODUCTION

Fungal contamination in agricultural crops, especially mycotoxigenic fungi and their toxic metabolites the mycotoxins, presents a significant global food safety challenge (1). These fungi are widely distributed in nature, encompassing hundreds of species in organic organisms and inorganic habitats or substrates. In a fungal community, different fungal species and even diverse strains of the same species interact and influence each other, affecting the structure and function of the entire fungal environment (2, 3). The analysis of fungal community composition and interaction, especially the fungal community diversity of harmful fungi, in various agricultural commodities is highly significant for food security (4).

Malt is an essential product derived from barley that displays excellent edible and medicinal properties for domestic and export markets (5–7). More notably, malt is the most important raw material for brewing beer, the second most popular alcoholic beverage consumed worldwide (8). A recent MarketsandMarkets report (9) indicated that the global malt extract and ingredients market stimulated by the growing demand for beer is expected to reach $20.4 billion by 2025. Unfortunately, fungal communities are naturally associated with barley during the malting procedure. This complex biological process involves many biochemical and physiological reactions that cause changes in the fungal community while affecting the quality and safety of malt and its end products in the value chain, such as beer (10, 11). More concerning is that some toxigenic fungi can biosynthesize mycotoxins during the production, storage, and transportation processes of malt and related products (12), causing significant risk to human health (13–15). Different processing methods were attempted to inhibit fungal growth and mycotoxin accumulation in the malt. Of these, the roasting treatment, with varied effects on fungal counts and mycotoxin production in foods, was verified (16, 17), resulting in two kinds of primary malt product, raw malt and roasted malt, that have circulated in the market for a long period with large import and export outputs. However, once food is contaminated with toxigenic fungi and mycotoxins, it is not fit for consumption, since the roasting process cannot completely eliminate fungal and mycotoxin contamination (18, 19). Therefore, regardless of the function of the malt, it is essential to monitor the fungal community diversity in real time, especially toxin-producing fungi, as well as mycotoxin residues. Prevention and control measures should be taken to ensure quality and safety and avoid economic losses.

Various conventional culture-dependent isolation and identification techniques are available and commonly applied to assess the fungal communities in food-based ecosystems (20–22). However, these methods are time consuming and labor intensive and are biased toward selective growth media, with less than 10% of the total number of fungal communities being identified (23). In addition, very few DNA sequences are obtained, limiting some important information regarding the fungal community structure (24). Therefore, developing a novel technique independent of culture separation is essential. With the rapid evolution of molecular biology and bioinformatics, molecular identification techniques like high-throughput sequencing (HTS), which is highly significant for the superior and inexpensive detection of rare taxa and fungal communities that cannot be identified by traditional culture methods, have increased the understanding of fungal community diversity and composition remarkably (25–27). However, the second-generation HTS technology can only be used for certain variable regions of bacterial 16S rRNA genes and fungal internal transcribed spacers (ITS) or 18S ribosomal DNA (rDNA), in which diverse biases in primer selection and short read lengths present the primary challenges (25). The selection of PCR primers for short-read-length amplifiers in different hypervariable regions affects the accuracy of detection for inferred microbiota communities and the sensitivity to certain taxa, making it difficult to compare microbiome diversity at the global level (28). In addition, second-generation sequencing of fungal communities usually occurs at the genus level (29), while identification cannot be accurately achieved at the species level due to limited variant information in the high-variation regions.

The third-generation single-molecule real-time (SMRT) sequencing technology combines the benefits of both ITS1 and ITS2 subregions to achieve high-throughput and full-length sequencing in the ITS region. The SMRT-based sequencing platform operated at the single-molecule level provides much longer read lengths (>20 kb) than the earlier generations and can directly obtain the sequence information of all variable regions in a short period and achieve accurate fungal community identification at the species level (30–32). Full-length SMRT sequencing can not only enhance the resolution of species identification but also avoid information loss and base mismatch to improve the accuracy of fungal composition classification and effectively characterize the fungal community structure. However, minimal studies are available that use this sequencing technology to investigate the fungal community diversity in food.

**FIG 1:**
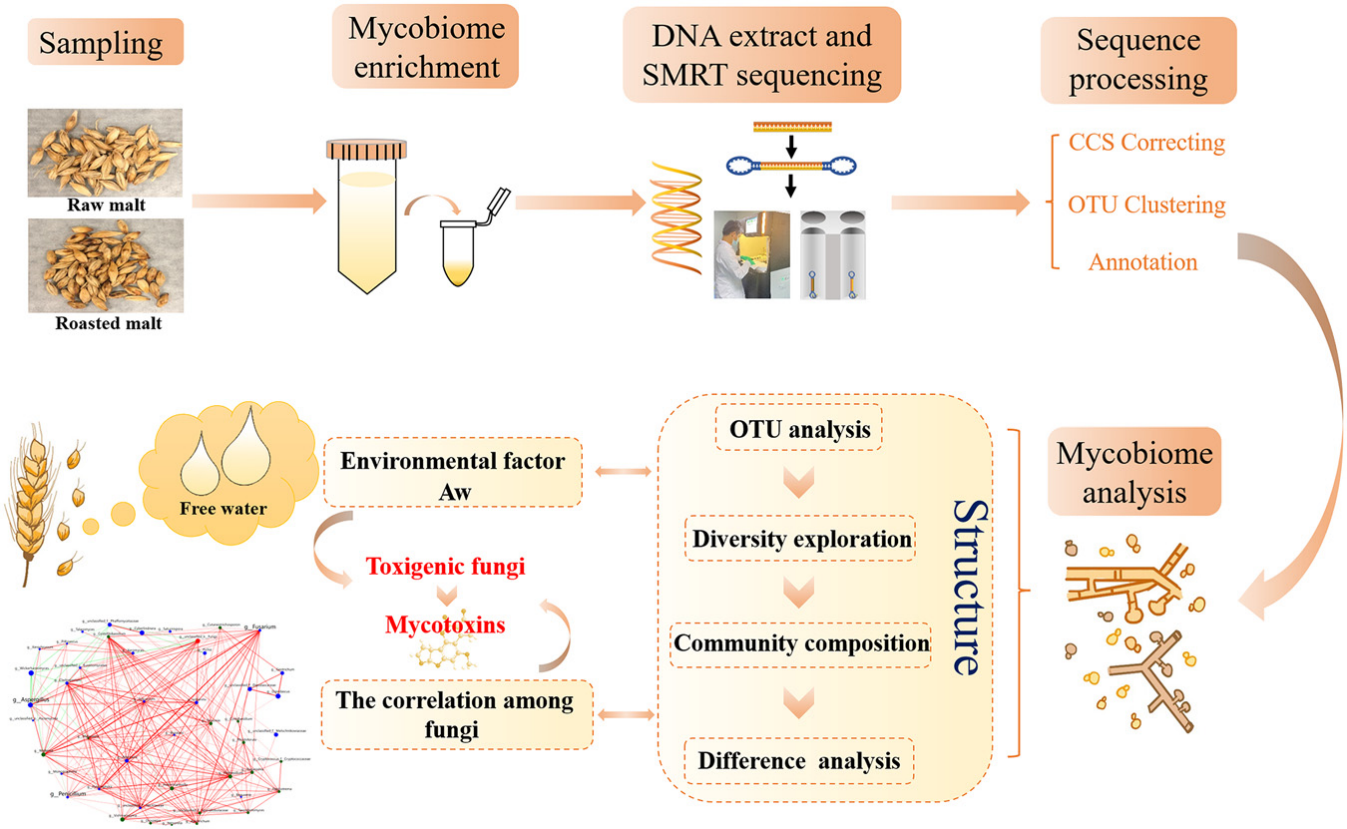
SMRT exploration of mycobiome diversity in raw and roasted malts.

Therefore, this study aims to achieve the following: (i) explore the fungal communities of raw and roasted malts from the main production areas in China by conducting PacBio-based full-length SMRT sequencing of the ITS region, with particular attention to mycotoxin-producing fungi belonging to the Aspergillus, Penicillium, and Fusarium genera; (ii) compare the mycobiome diversities and compositions in raw and roasted malts; (iii) molecularly identify the fungal species enriched in the malts; (iv) determine the water activity (a_w_) and quantify mycotoxins, including aflatoxin B_1_ (AFB_1_), AFB_2_, AFG_1_, and AFG_2_, ochratoxin A (OTA), and zearalenone (ZEN), using ultrafast liquid chromatography tandem mass spectrometry (UFLC-MS/MS); (v) explain the relationship between the identified fungi and the mycotoxins produced; and (vi) assess the safety of the raw and roasted malts (Fig. 1). The results provide a powerful basis for subsequent functional analysis of the mycobiome and early risk warning and a reference for the quality and safety control of malt. This is the first study that explores mycobiome diversity in edible and medicinal malt using the third-generation SMRT technology, allowing evaluation of its application prospects in the future.

## RESULTS AND DISCUSSION

### Evaluation of the sequencing sample size and depth.

The SMRT sequencing was used to obtain a total of 766,383,003 bases and 1,310,563 optimized sequences. All the optimized sequences were between 300 bp and 900 bp long, with an average length of 584 bp, while those of ITS1 or ITS2 were usually between 250 bp and 400 bp during second-generation HTS sequencing. Therefore, the full-length SMRT sequencing of ITS produced much longer sequences, resulting in finer taxonomic resolution and reduced amplification of dead fungi (25). To eliminate the influence of differences in the amount of optimized sequences generated from each malt sample, the data volume of all samples was randomly extracted to the same data volume according to the fewest sequences, generated by sample M9 (44,784), for subsequent analysis. After operational taxonomic unit (OTU) clustering and annotation, a total of one domain, one kingdom, six phyla, 23 classes, 56 orders, 120 families, 188 genera, 333 species, and 780 OTUs were measured.

**FIG 2:**
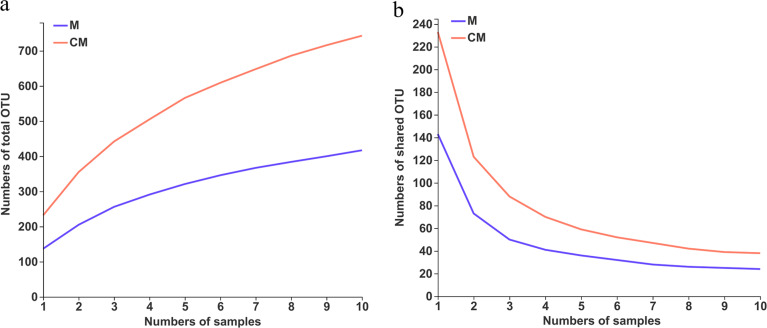
(a) Pan-OTU and (b) core OTU curves.

First, pan- and core species analyses were performed to evaluate whether the sample size of this sequencing process was sufficient. Pan- and core OTUs were used to describe the changes in the numbers of total species and core species after increasing the sample size. The pan-OTUs were the sum of OTUs contained in all samples, which was used to observe the increase in the total number of OTUs after increasing the sample size. The core OTUs referred to the number of common or shared OTUs in all samples, which was applied to monitor the decline in the number of OTUs common to all samples with an increase in sample size. As indicated by the results in Fig. 2a, the number of OTUs increased with an increase in sample size. In group M (raw malt samples), the measured number of total OTUs increased sharply when the sample size increased from 1 to 5. However, when the sample size exceeded 5, the number of total OTUs began to increase slowly. When the sample size reached 10, the pan-OTU curve was almost parallel to the *x* axis, indicating that the sample size in group M was sufficient. However, in group CM (roasted malt samples), the slope of the pan-OTU curve was much steeper than the slope in group M, even when the sample size reached 10. These findings indicated that the number of total OTUs continued to increase as the sample size in group CM continued to rise. However, this increasing trend was not obvious. A minimal increase in the number of pan-OTUs was evident after further increasing the sample size. However, in groups M and CM (Fig. 2b), the core OTU curves began to drop sharply with an increase in the sample size, gradually becoming parallel to the *x* axis when the sample size reached 5. The number of shared OTUs barely changed as the sample size continued to increase. In conclusion, when analyzing the fungal community diversity under the same environmental conditions in the same group, the core OTUs should receive more attention. Considering the cost and practicality of analysis, a sample size of 10 for the raw and roasted malt samples in each group selected in this study was sufficient for subsequent analysis.

**FIG 3:**
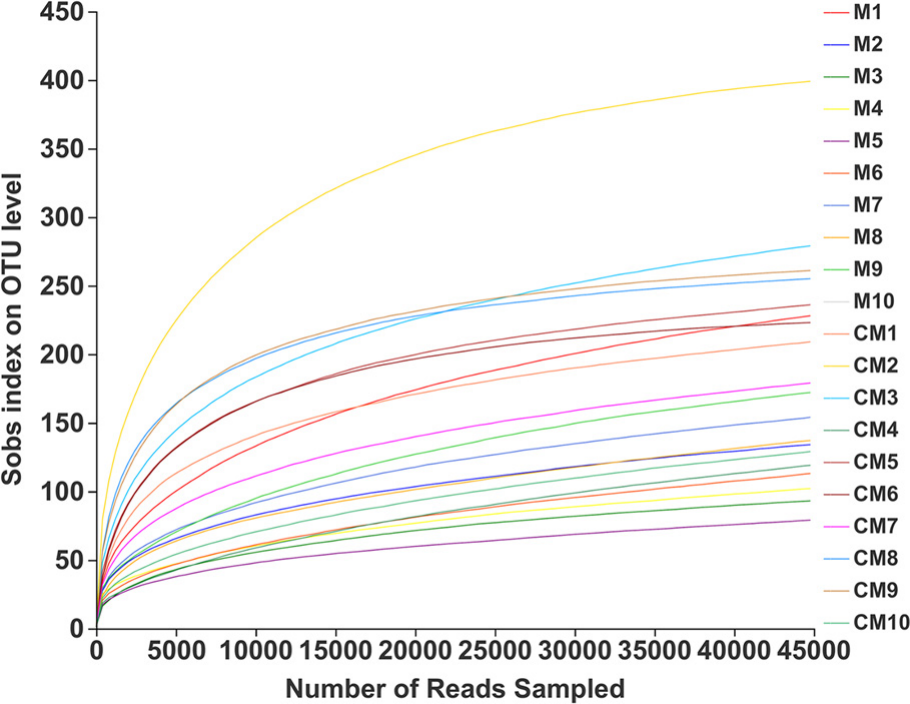
Rarefaction curves of 20 raw and roasted malt samples. Sobs, number of species observed.

The sequencing depth was evaluated according to the rarefaction curves, which were constructed by randomly sampling the number of sequences and their corresponding OTUs (*S*_obs_), with the *S*_obs_ index reflecting the actual numbers of species observed in the samples. As shown by the results in Fig. 3, the *S*_obs_ indexes of all the samples at the OTU level increased rapidly with increased numbers of sequences at the beginning, while this trend declined when the sequencing number reached 8,000. The rarefaction curves tended to be flat when the sequencing number exceeded 20,000. This implied that the sequencing depth was reasonable and more sequencing data would only generate a few new species. Combined with the optimized numbers of sequences of the 20 samples, listed in Table 1, it can be concluded that the sequencing number of each sample far exceeded 20,000, while the sequencing depth was sufficient for subsequent analysis.

**TABLE 1:** Detailed sample information

Group, sample^*a*^	a_w_^*b*^	Mycotoxin detected^*c*^	No. of sequences
M			
M1	0.643	—	50,367
M2	0.640	—	60,907
M3	0.640	—	72,818
M4	0.554	—	60,486
M5	0.760	ZEN	56,475
M6	0.627	—	74,311
M7	0.571	—	76,486
M8	0.561	—	64,266
M9	0.612	—	52,811
M10	0.601	—	61,682
C			
CM1	0.580	—	77,003
CM2	0.625	—	60,632
CM3	0.686	—	61,382
CM4	0.770	—	67,745
CM5	0.489	—	68,420
CM6	0.620	—	77,159
CM7	0.544	—	69,214
CM8	0.262	ZEN	61,791
CM9	0.216	ZEN	70,023
CM10	0.546	—	66,585

a The origin of all samples was Hebei Province.

b a_w_, water activity.

c Samples were analyzed for six mycotoxins, AFB_1_, AFB_2_, AFG_1_, AFG_2_, OTA, and ZEN. —, mycotoxins not detected; ZEN, only ZEN detected.

### Diversity analysis of the fungal communities in the malt samples.

Alpha diversity analysis reflects the richness and diversity of the fungal community in a single sample. This study used four statistical analysis indexes, namely, ACE (abundance-based coverage estimator), Shannon, Shannon evenness, and Good’s coverage, to reflect the richness, diversity, evenness, and coverage of the fungal communities in each malt sample. According to the data presented in Table 2, the ACE indexes of all 20 samples exceeded 150, while the ACE indexes of sample M8 (343.303554) in group M and sample CM2 (423.723694) in group CM were significantly higher than those of the other samples, indicating that the two raw and roasted samples exhibited the highest community richness and contained the largest number of species. Contrarily, samples M3 and M4 and samples CM6 and CM7 presented the lowest ACE indexes, implying lower fungal community abundance and fewer species. Furthermore, the Shannon index of sample CM2 was also the largest, while that of sample M10 was the smallest, indicating that sample CM2 displayed not only the highest fungal community richness but also the highest fungal community diversity. Sample M10 displayed the lowest fungal community diversity of all 20 samples. In addition, samples M10 and CM2 presented the lowest and highest Shannon evenness indices, respectively, in terms of fungal community evenness. Coverage refers to the coverage of each sample library, reflecting whether the sequencing results represent the actual fungal situation in a sample. A higher coverage value means a higher probability of detecting a sequence in the sample. The coverage indexes of all the samples exceeded 0.99 (Table 2), indicating that SMRT sequencing allowed high community coverage and could represent the true fungal communities in these samples.

**TABLE 2:** Alpha diversity indices in all malt samples

Sample	Value for indicated index			
	ACE	Shannon	Shannon evenness	Good’s coverage
M1	305.398231	1.851993	0.341124	0.998479
M2	199.429087	2.395371	0.489143	0.999281
M3	157.62531	1.732686	0.381879	0.999425
M4	186.769637	2.535983	0.548516	0.999255
M5	188.856234	1.978152	0.452673	0.999337
M6	298.523699	1.453476	0.309044	0.999203
M7	267.70738	2.530907	0.503204	0.99926
M8	343.303554	1.603287	0.32643	0.999029
M9	234.126421	2.571403	0.499545	0.99875
M10	278.332743	1.044541	0.223769	0.999045
CM1	293.728281	2.307656	0.429881	0.999217
CM2	423.723694	3.900979	0.651086	0.999088
CM3	349.880331	2.675367	0.475083	0.998717
CM4	290.875916	1.415505	0.296583	0.999063
CM5	277.314152	2.642196	0.48341	0.999396
CM6	249.659146	1.646463	0.305575	0.999658
CM7	258.351905	2.374862	0.457105	0.999175
CM8	285.127037	3.0413	0.547953	0.999452
CM9	287.184948	3.158172	0.567635	0.999557
CM10	290.317575	2.384645	0.490199	0.998879

**FIG 4:**
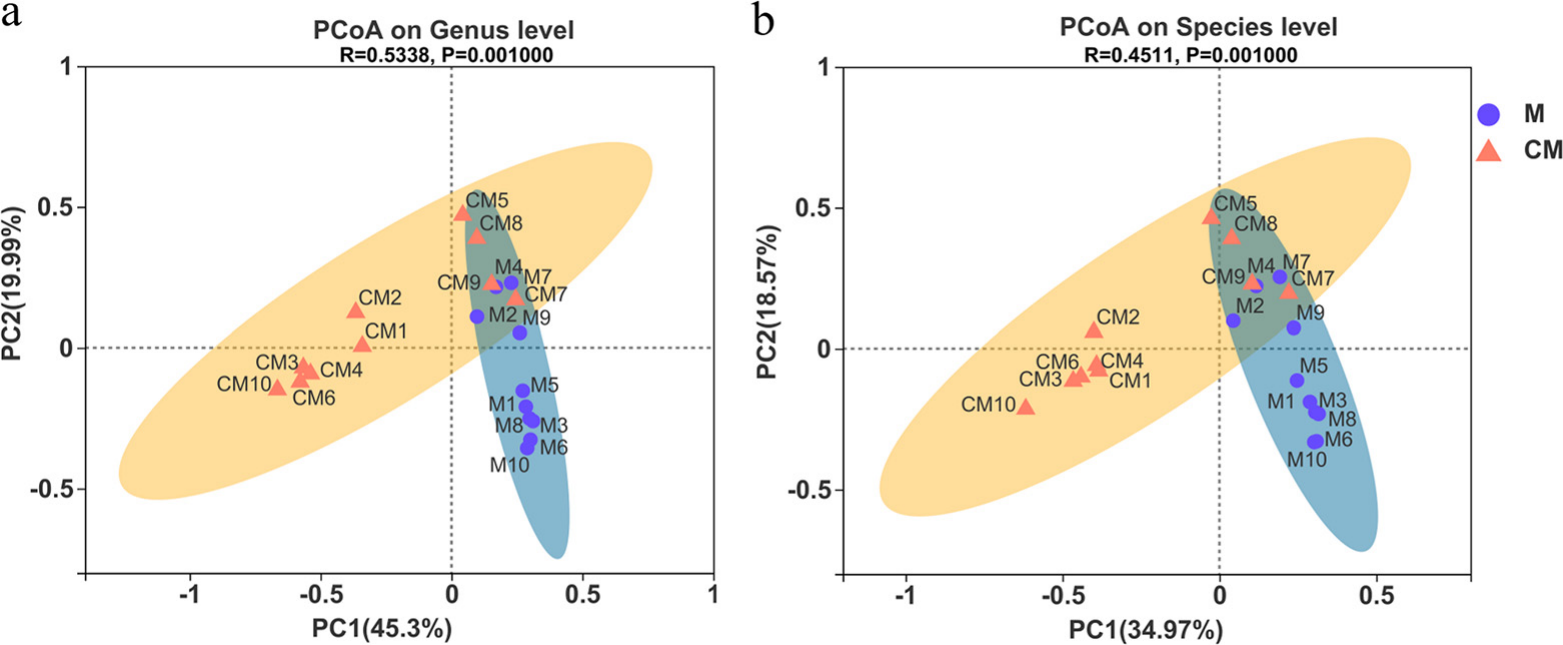
PCoA graphs based on Bray-Curtis distance at the genus (a) and species (b) levels.

Alpha diversity was used to analyze the fungal community diversity in a single sample. However, this method was far from sufficient during practical analysis to successfully accomplish this task. Comparing the community differences between the diverse environments/samples/groups is more conducive to studying fungi. However, analysis via traditional culture and separation methods is often performed at the genus, species, and strain levels (33, 34). Inversely, the current level of research could not distinguish and identify fungal strains during sequencing. Although the third-generation SMRT sequencing technique exhibits higher resolution than second-generation HTS sequencing at the species level, the fungal annotation databases require improvement and expansion, especially at the species level. Therefore, beta diversity analysis was conducted to compare the similarities between the species diversities of these samples. Considering the accuracy of the annotation results, the beta diversity of the samples was analyzed at both the species and genus levels to increase the accuracy of the findings. Principal-coordinate analysis (PCoA) was performed to compare the differences between the species compositions of the two groups. The PCoA graph was based on Bray-Curtis distance at the genus and species levels, as shown by the results in Fig. 4a and b, respectively.

At the genus level, principal coordinate 1 (PC1) and PC2 accounted for 45.3% and 19.99% of all the original information, with a sum of 65.29%, >50% (Fig. 4a), indicating that the two principal axes exhibited a high degree of explanation for the differences between the fungal compositions of the samples. Therefore, the subsequent analysis was based on these two main axes. The samples of groups M and CM were well clustered in their respective confidence ellipses, presenting relatively similar genus compositions. The intersection of the two confidence ellipses included samples CM5, CM7, CM8, CM9, M2, M4, and M7, showing little difference between the respective genus compositions. In group M, samples M1, M3, M5, M6, M8, and M10 were extremely close to each other, indicating fungal composition similarities at the genus level. As for the remaining samples, samples M4 and M7 were the closest together but deviated furthest from the other samples in group M, which might be because the external conditions during the growth, treatment, transportation, and storage processes affected their fungal community compositions. Similarly, in group CM, samples CM3, CM4, CM6, and CM10 exhibited similarities in fungal communities at the genus level. The compositions of samples CM5 and CM7 were more unique than those of the other samples.

At the species level (Fig. 4b), PC1 and PC2 accounted for 34.97% and 18.57%, respectively, of all the original information, displaying similar results consistent with those at the genus level (Fig. 4a).

### The fungal community compositions in the malt samples.

**FIG 5:**
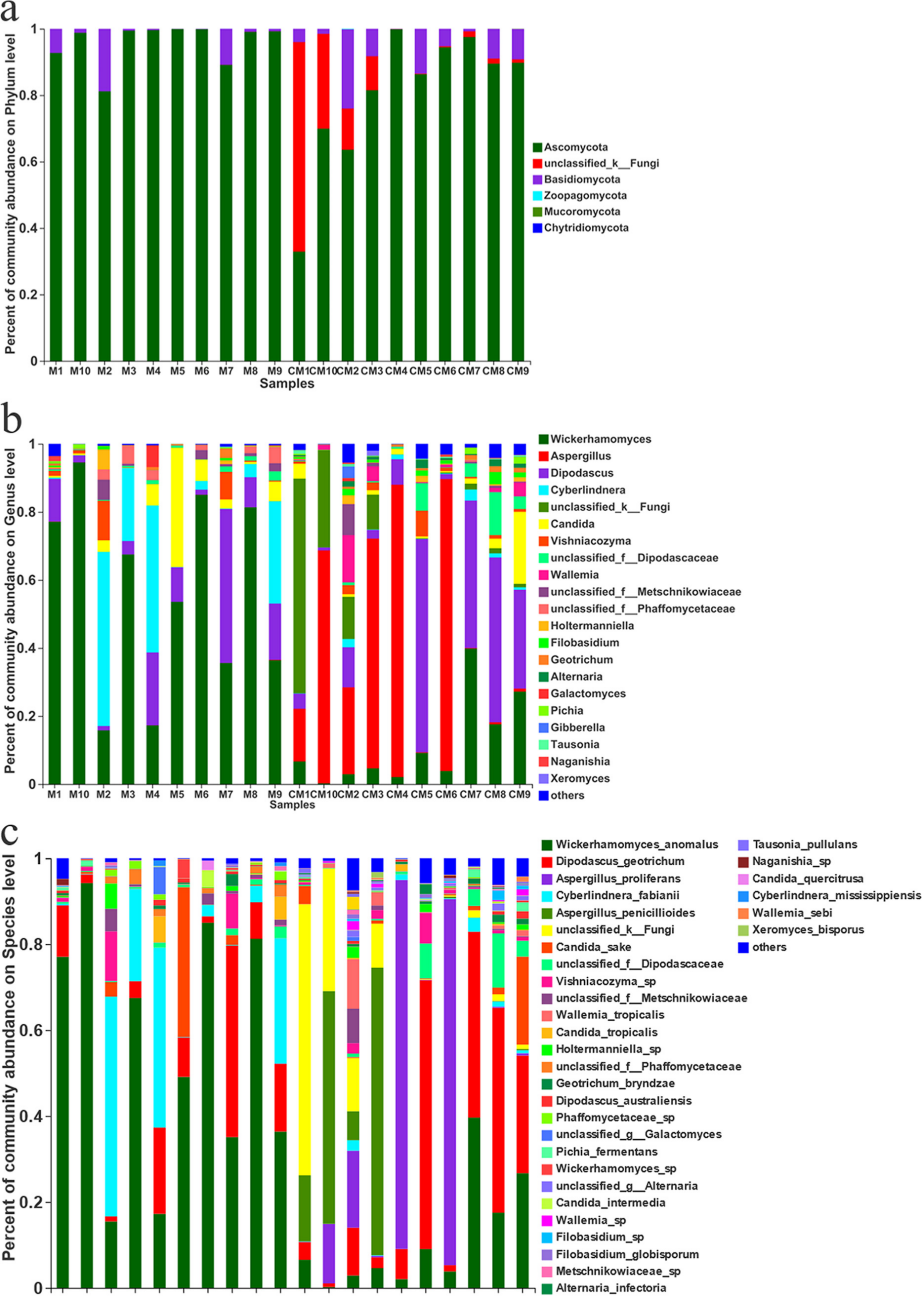
Bar diagrams of fungal composition at the phylum (a), genus (b), and species (c) levels.

Extensive analysis of the specific taxonomic compositions in malt can allow visual displays of the fungal community structures, providing a foundation for the subsequent analysis of fungal community differences, associations, evolution, and function prediction. Therefore, the taxonomic composition of each malt sample is discussed in detail, particularly emphasizing the dominant species and toxigenic fungi. The diversity of the fungal communities was closely related to the compositions of specific taxa. A total of one kingdom, six phyla, 23 classes, 56 orders, 120 families, 188 genera, and 333 species were identified in 20 samples via SMRT sequencing. The bar diagrams of the fungal community compositions at the phylum, genus, and species levels are presented in Fig. 5.

As shown in Fig. 5a, *Ascomycota* and *Basidiomycota* were the most dominant phyla, with relative abundance values of 32.88 to 99.93% and 0.07 to 23.86%, respectively. *Ascomycota* displayed obvious advantages in all the samples. At the genus level (Fig. 5b), Wickerhamomyces, Aspergillus, Dipodascus, Cyberlindnera, and Candida were the dominant fungi, with relative abundance values of 0.21 to 94.53%, 0 to 85.92%, 0.87 to 62.69%, 0.02 to 51.15%, and 0.03 to 35.01%, respectively. Aspergillus, Penicillium, and Fusarium were the most common fungal genera with the capacity to produce mycotoxins. For instance, toxigenic fungi of the Aspergillus and Penicillium genera produced aflatoxins and ochratoxins, while Fusarium generated ZEN and fumonisins (35), commonly reported to contaminate barley, malt, and related products (36, 37). Therefore, these fungal genera should be monitored continuously, especially some specific toxigenic species or strains. Further analysis of the bar diagrams of the fungal community compositions at the species level (Fig. 5c) indicated that Wickerhamomyces anomalus (0.21 to 94.22%), Dipodascus geotrichum (0.84 to 62.59%), Aspergillus proliferans (0 to 85.88%), Cyberlindnera fabianii (0.02 to 51.13%), Aspergillus penicillioides (0 to 66.94%), and Candida sake (0 to 34.85%) represented the dominant fungal species. Aspergillus minisclerotigenes, Aspergillus sclerotiorum, Fusarium poae, Candida tropicalis, and Trichothecium roseum were the main toxigenic and pathogenic fungi detected in the malt samples, three of which (samples M5, CM8, and CM9) were ZEN positive (Table 1). Notably, Aspergillus minisclerotigenes can produce aflatoxins, while some Aspergillus sclerotiorum strains can generate OTA (38, 39), indicating that the presence of toxigenic fungi in the malt did not necessarily result in the production and accumulation of mycotoxins. However, the absence of visible molds does not guarantee the absence of mycotoxins in food. Mycotoxins are not produced systemically, and the fungi capable of producing mycotoxins may not do so in, for example, roasted barley malts. However, toxigenic fungi generally do not exist alone, they grow in the environment or a matrix as a colony and engage in complicated interactions. Different fungal species, even subspecies, interact with each other (inhibitory or synergistic effects), the environment, or the matrix. There is consensus that mycotoxin production and contamination levels are highly dependent on environmental conditions, such as ambient temperature, water content, a_w_, pH, relative air humidity, and oxygen content, as well as the food substrate itself (nutrient composition), physical damage, and the presence of fungal spores. The optimal nascence temperature range for most mycotoxins is 20 to 30°C, while the a_w_ should exceed 0.7. Different food substrates contain various nutritional compositions. Studies have shown that certain fungi prefer specific types of foods. For example, Fusarium is more likely to infect cereals and relevant products rich in starch, leading to the production of and pollution by mycotoxins, such as deoxynivalenol (DON), ZEN, and fumonisins (40). After mycotoxins are produced, they may also be degraded by other fungi, such as Rhizopus oryzae (41), reducing the risk to the matrix and the environment. A previous study (27) found that an OTU sequence annotated in the UNITE 8.0 database corresponded to Rhizopus arrhizus, while Rhizopus oryzae was annotated in the NCBI (National Center for Biotechnology Information) database. Contrarily, the Rhizopus arrhizus sequence in the UNITE 8.0 database was also detected during this sequencing process. The BLAST results of the OTUs corresponding to the species in NCBI also referred to Rhizopus arrhizus. This demonstrated the reliability of third-generation SMRT sequencing at the species level and showed that it could characterize the fungal community structure in malt samples more accurately than the second-generation HTS technology.

**FIG 6:**
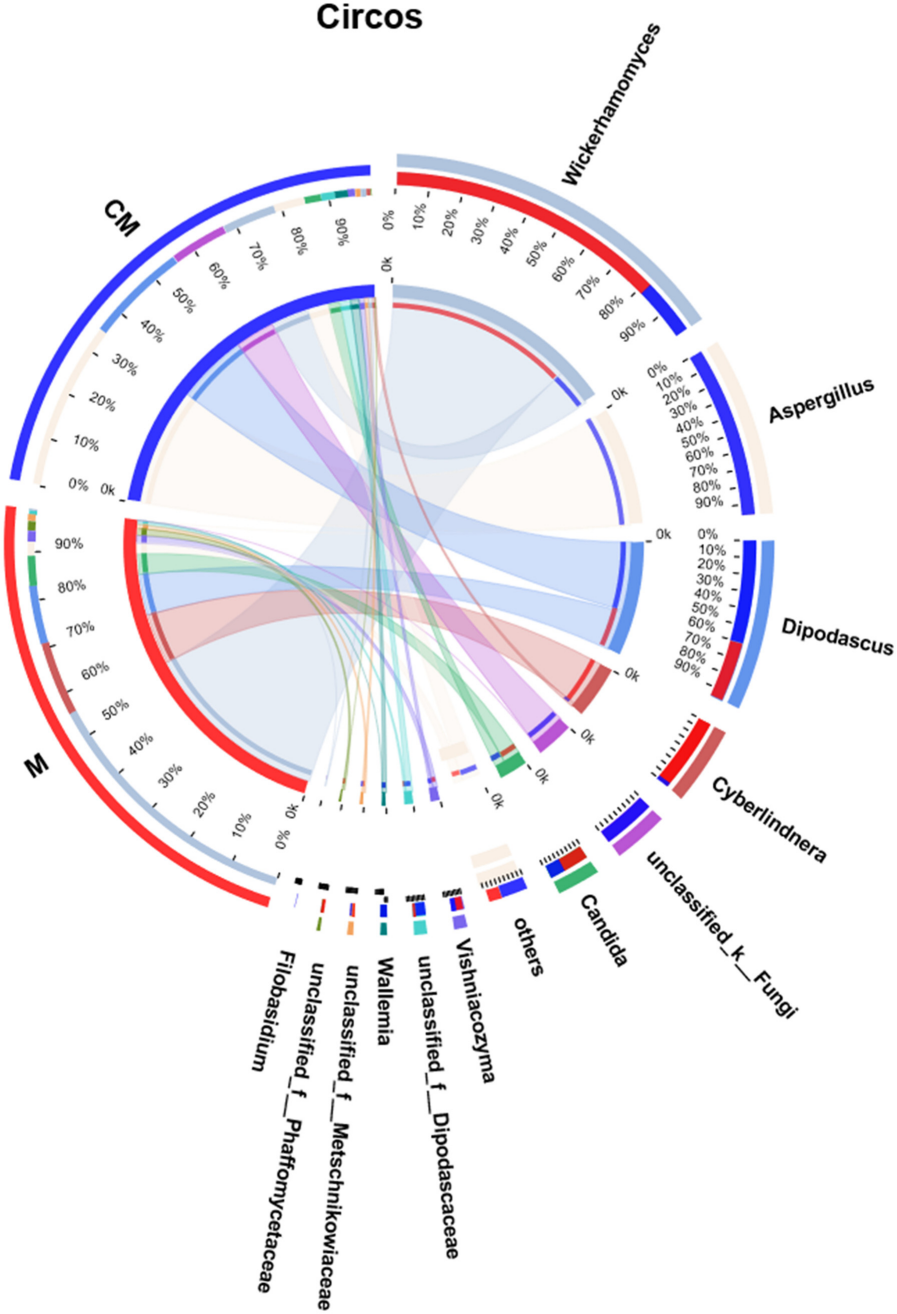
Circos diagram revealing the relationships between malt samples and fungi at the genus level.

To further visualize the proportions of the dominant genera in the malt samples of the different groups, a Circos diagram at the genus level was created, as shown in Fig. 6. Wickerhamomyces (56%), Cyberlindnera (15%), Dipodascus (12%), and Candida (6.1%) were the dominant genera in group M, while Aspergillus (35%), Dipodascus (21%), Wickerhamomyces (11%), and Candida (3.5%) represented the most abundant genera in group CM. Wickerhamomyces displayed obvious advantages in group M and Aspergillus in group CM. Compared with group M, the malt samples in group CM only received one additional roasting treatment. However, significant differences were evident between the fungal communities of the raw malt samples in group M and the roasted malts in group CM. Aspergillus produces mycotoxins like AFs and OTA, while Candida can cause serious diseases. As shown in the right-hand portion of Fig. 6, the relative abundance of Aspergillus in the roasted malt samples in group CM exceeded that in the raw samples in group M, while the relative abundance of Candida in group CM was lower than that in group M. Some Aspergillus species reportedly displayed a certain degree of heat resistance (42), which might be one of the reasons why Aspergillus represented the dominant fungi in the CM group. Unlike pathogenic fungi like Candida, the production of mycotoxins from toxigenic fungi was affected by many internal and external factors. Although Aspergillus was the dominant fungus in the CM group, Rhizopus, another dominant fungus, inhibited the growth and reproduction of Aspergillus. In addition, the complicated interaction between different Aspergillus species, as well as some nontoxigenic Aspergillus fungi, restricted the proliferation of toxigenic Aspergillus fungi (43). Therefore, the roasting treatment may facilitate structural and compositional changes by promoting or repressing certain fungal communities, further lowering the risk due to some toxigenic fungi in the roasted malts.

### Quantitative PCR (qPCR) validation for detected fungi.

To validate the detected fungi, Aspergillus proliferans, Aspergillus sclerotiorum, and Candida tropicalis were selected as the representative dominant fungus, toxigenic fungus, and pathogenic fungus, respectively. Moreover, three pairs of specific primers were designed according to the OTU sequences corresponding to these three species (Table S1). The DNA was successfully extracted from the tested samples (CM6, CM8, and M4) containing the detected fungi. The spectrophotometric results showed that the optical density (OD) ratio values ranged from 1.91 to 1.99 at 260 nm to 280 nm and from 1.56 to 2.24 at 260 nm to 230 nm. The DNA concentration of each sample was 128.5 to 302.9 ng/μL, further meeting the experimental requirements. To further optimize the experimental conditions, the gradient PCR experiment for each primer was performed in a temperature range of 53 to 57°C, revealing that the amplification effect at 55°C was the most suitable; thus, this was selected as the appropriate annealing temperature for the subsequent experiments. As shown by the results in Fig. S1, all three pairs of specific primers could amplify the target DNA fragments, presenting a standard S-shaped amplification curve, which differed significantly from the cycle threshold (*C_T_*) value of the control hole without the template. As shown by the results in Table S2, the average *C_T_* values of the three fungi (Candida tropicalis, Aspergillus proliferans, and Aspergillus sclerotiorum) were 19.02, 25.66, and 27.61, respectively, confirming the reliability and accuracy of the sequencing results.

### A comparison between the fungal communities of the raw and roasted malts.

**FIG 7:**
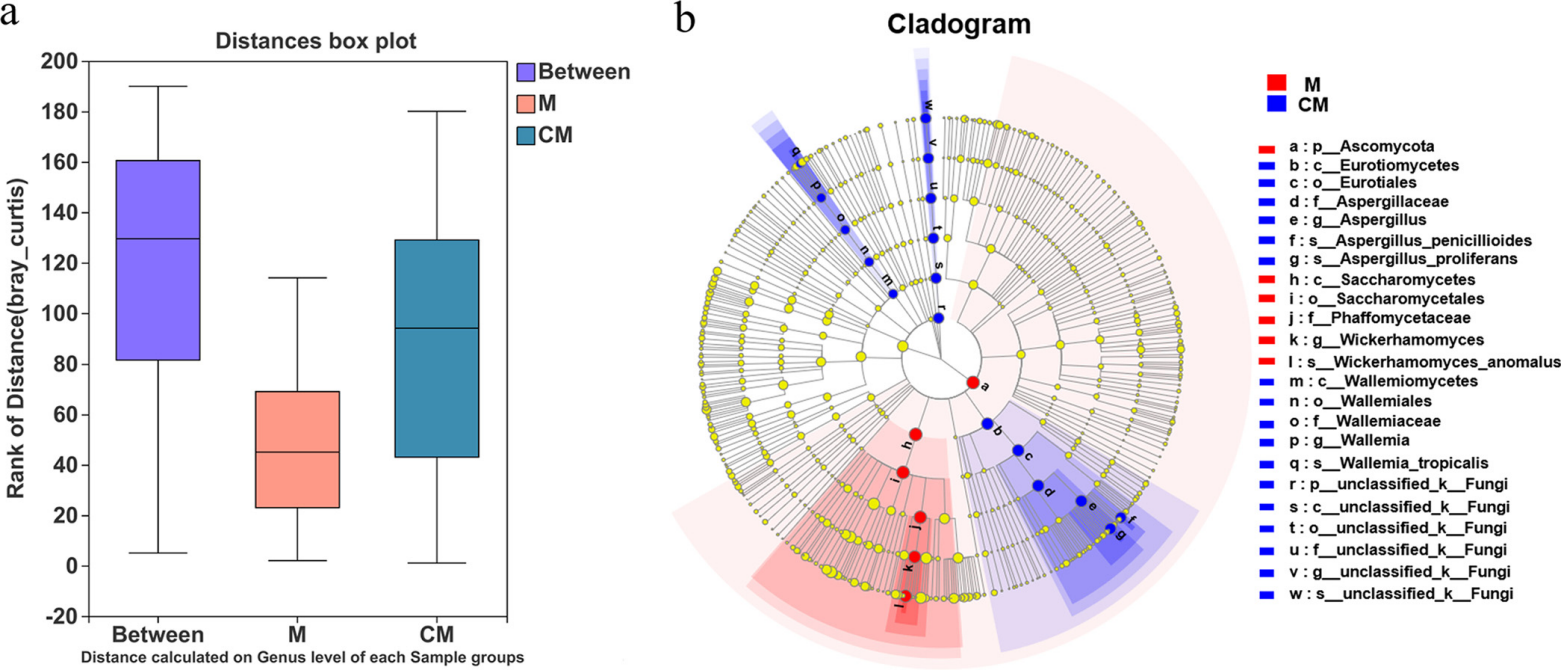
(a) ANOSIM box plot based on Bray-Curtis distance. (b) LEfSe hierarchical cladogram of multilevel taxa. The nodes marked by yellow circles indicate no significant differences between groups M and CM. The red and blue nodes represent species displaying substantial differences in abundance in the M and CM groups, respectively. The diameter of a node illustrates the importance of the difference.

Raw and roasted malts, also known as unbaked and baked malts, are highly popular with consumers. Roasted malt is obtained by simply roasting raw malt, changing the original flavor and preventing contamination by mold and insects. However, roasting cannot kill or completely eliminate existing molds. This study investigated the differences between the fungal community structures of raw and roasted malts. As shown by the results in Fig. S2, 404 OTUs were identified in group M and 734 in group CM, 358 of which were shared. Group M only had 46 unique OTUs, while group CM exhibited 376. In general, one OTU represented one species (44). Therefore, group CM exhibited more unique species than group M, which might be introduced via the environment during the roasting process. Although roasting may not destroy mycotoxins or fungal DNA, most roasting conditions successfully eliminate most molds, certainly including Candida tropicalis and related yeasts. However, these fungi were detected in the roasted malts, which were likely contaminated after the roasting process. The analysis of similarity (ANOSIM) box plot based on Bray-Curtis distance (Fig. 7a) showed that the differences between the two groups were more significant than those within the groups, while the median ranks of distance were as follows: between groups > group CM > group M. Statistically significant differences were evident between the two groups (*R *= 0.5338, *P* < 0.01).

Next, the exact differences between the sample groups were discussed. The Wilcoxon rank-sum test bar plots at the genus level (Fig. S3) showed that only 2 genera with significant differences were identified in group M and 13 in group CM. These findings were consistent with the Circos graph presented in Fig. 6. In addition, the three mycotoxin-producing genera, namely, Aspergillus, Fusarium, and Penicillium, were mainly distributed in group CM, indicating that the roasted malt samples presented a higher risk than the group M samples. Further analysis should be conducted in combination with relevant environmental factors.

Linear discriminant analysis effect size (LEfSe) analysis was performed to explore the statistically different biomarkers between the two groups and estimate the impact of the abundance of each component (species). In the LEfSe hierarchical cladogram of the multilevel taxa (Fig. 7b), the nodes marked by yellow circles indicate no significant differences between groups M and CM. The red and blue nodes represent species displaying substantial differences in abundance in the M and CM groups, respectively. In group CM, besides unclassified species, the other three blue nodes correspond to Aspergillus proliferans, Aspergillus penicillioides, and Wallemia tropicalis. In group M, Wickerhamomyces anomalus was denoted the most statistically different species, and the diameter of its corresponding red node was larger than those of other biomarkers, illustrating that it was the most important species causing the differences between groups M and CM. In addition, although Aspergillus proliferans, Aspergillus penicillioides, and Wickerhamomyces anomalus belong to the same phylum—*Ascomycota*—evolutionarily, they were regarded as the dominant species or biomarkers from different groups.

### Analysis of the correlations of fungal communities with environmental factors.

It is well known that the reproduction and metabolism of fungi and fungal communities depend on the surrounding environmental conditions, such as nutritional composition, temperature, oxygen, and a_w_. The same is true for fungal communities in malt. Starch, amino acids, and other chemical components in malt can provide carbon and nitrogen sources for fungi. Similarly, the a_w_ in malt influences the growth, reproduction, and metabolism of the fungi, facilitating structural changes in the fungal communities in turn. Here, a_w_ was the most relevant factor in the roasted malt. To further explore the differences between the fungal community structures in the raw and roasted malts, the a_w_ was considered a vital environmental factor when determining the correlations via redundancy analysis (RDA).

**FIG 8:**
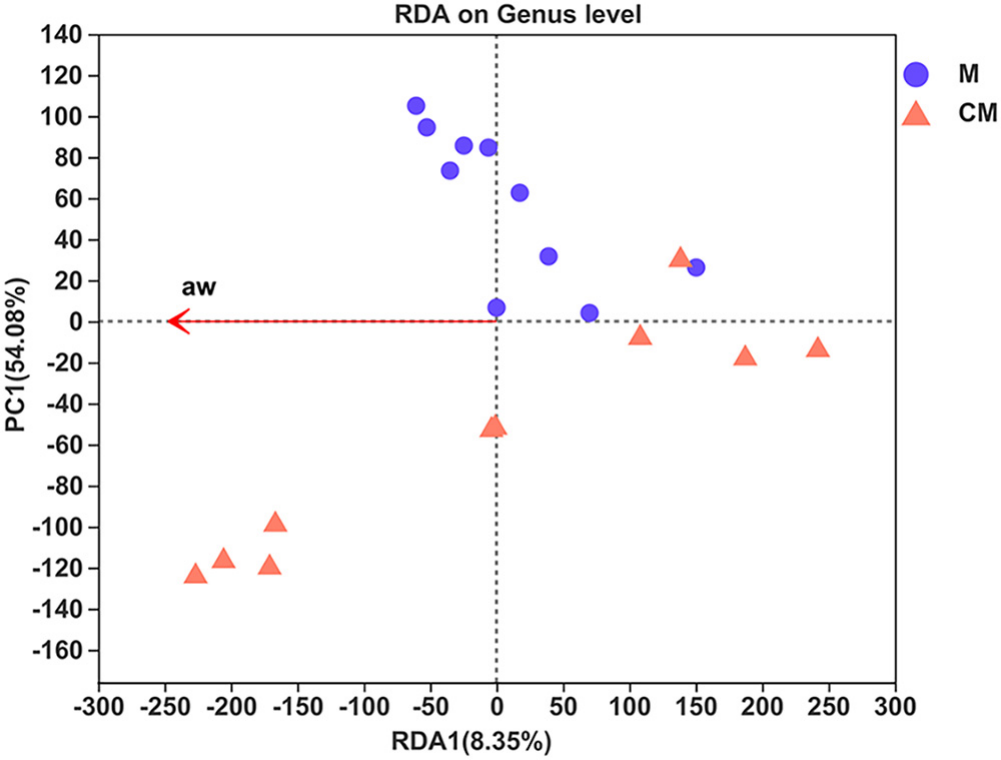
RDA graph at the genus level. The longer the red arrow, the greater the impact.

In the RDA diagram (Fig. 8), the red arrow representing the environmental factors is long, indicating the significant impact of a_w_ on the fungal communities. The projection points of these 20 samples on the red arrow show that a_w_ influenced the distribution of the fungal communities of both the M and CM groups. Compared with group M, most of the projection points of the corresponding malt samples in group CM are far from the origin, indicating a more significant impact by a_w_ on the fungal community distribution in the roasted malt samples.

As shown by the results in Table 1, the measured a_w_ values were mostly concentrated between 0.55 and 0.65. Only sample M5, which was ZEN positive, exhibited an a_w_ value as high as 0.760. This confirmed that high a_w_ conditions were more conducive to the growth, reproduction, and metabolism of toxigenic fungi like Fusarium, as well as the production of the secondary metabolite ZEN (45). In contrast, the a_w_ value distribution in group CM was more dispersed, ranging from 0.21 to 0.77, which could be due to the continuous variation in a_w_ with ambient humidity. However, none of the six target mycotoxins were detected in sample CM4, which displayed the highest a_w_ value of 0.770. This may be because the toxigenic fungi on the surface of the malt were inhibited or killed during the roasting process. This also verified that, without the introduction of new toxigenic fungi, mycotoxins were not produced even under high-a_w_ conditions. ZEN was detected in samples CM8 and CM9, which displayed the lowest a_w_ values, which might be attributed to the existing toxigenic fungi and ZEN present before roasting. Although roasted, the mycotoxins were not completely degraded or eliminated and remained.

### Correlation analysis of the fungi.

**FIG 9:**
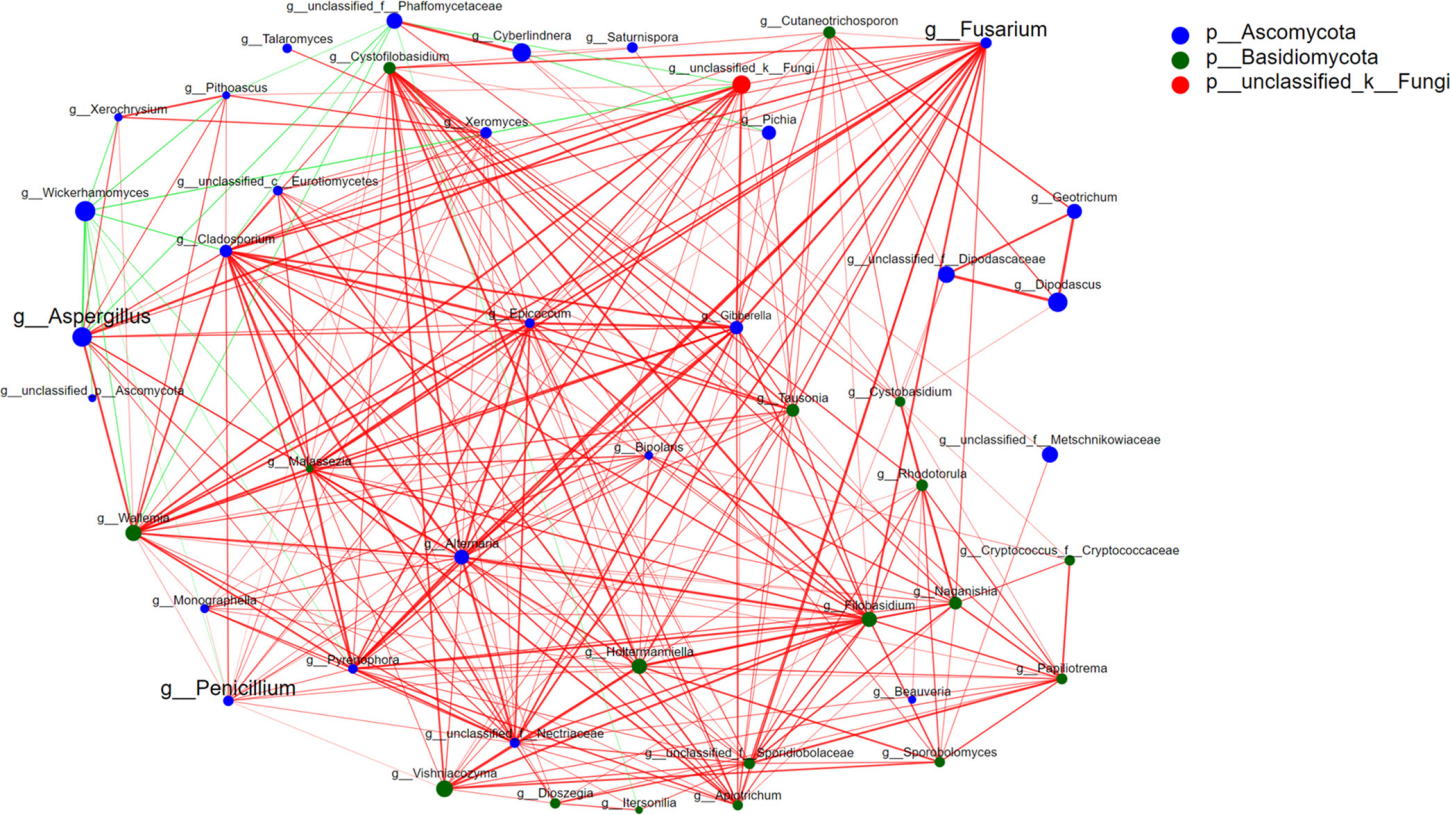
Spearman correlation network diagram of the relative abundances of the top 50 genera to explore the correlations among fungi. Red lines represent positive correlations of the fungi, green lines denote negative correlations, and thickness indicates degree of correlation.

Here, the correlations between the identified fungi are discussed to determine their mechanisms. Figure 9 shows a Spearman correlation network diagram of the relative abundances of the top 50 genera. It was drawn based on the correlations between the genera to obtain the interactions of the taxa in the same environment and further explain the mechanism of the formation of phenotypical differences between groups M and CM. The red lines in Fig. 9 represent positive correlations of the fungi, the green lines denote negative correlations, and the thickness indicates degree of correlation.

The relative abundances of the top 50 genera displaying various positive and negative correlations are presented in Fig. 9. For most genera, a genus of fungi was relevant to more than three other genera, with mostly positive correlations. Regarding the toxigenic fungi, more than 10 genera were related to Aspergillus, and only two exhibited negative relationships. Wickerhamomyces displayed the most significant negative correlation with Aspergillus. Similarly, more than 10 genera were correlated with Penicillium, while Wickerhamomyces was also negatively correlated. Another ZEN-producing fungus, Fusarium, exhibited a positive correlation with most of the fungi identified, including Aspergillus, presenting substantial symbiotic relationships. The fungus Wickerhamomyces, which was negatively correlated with Aspergillus and Penicillium, likely inhibited the growth of the two toxigenic fungi to reduce their toxicity.

Therefore, in practice, these toxigenic fungi, as well as other related fungi, especially those with a negative correlation that could be due to inhibition of the growth of toxigenic fungi, should be monitored to ensure the quality and safety of malt and malt-derived products.

### Safety assessment of the raw and roasted malts.

The identified fungal communities, especially some toxigenic fungi, produced mycotoxins, seriously threatening the safety of the malt and related products. Mildew is caused by the mass reproduction and metabolism of fungal communities, including filamentous fungi like Aspergillus, Penicillium, and Fusarium. The mildewing process is often accompanied by the production of mycotoxins. Therefore, directly observing the mildewing process on the malt allowed rapid monitoring of fungal growth to evaluate the safety of the malt.

Here, the changes in appearance due to mildew on the surfaces of the 20 raw and roasted malts in the culture dishes were monitored for 5 days at room temperature. As shown by the results in Fig. S4, no mildew was observed during the first 2 days. However, after 3 days, all of the raw malt samples in group M exhibited obvious mildew, especially on the surfaces of samples M1, M8, and M9. In group CM, six roasted samples, CM1, CM2, CM4, CM6, CM7, and CM10, developed mold, with CM4 the most significantly affected. More obvious mildew was observed in group M than in group CM. At 4 days, nine raw malt samples were severely affected by mold, while sample M4 was slightly moldy, and all the roasted malt samples except CM5 and CM9 were moldy. At 5 days, all the roasted malt samples were moldy. Significant mildew was observed on the surface of sample CM5, followed by samples CM8 and CM9. The raw malts in group M were more prone to mildew and less safe than the roasted malts in group CM, indicating that the roasting may decrease the a_w_ values and reduce the incidence of mold and contamination by toxigenic fungi to improve the safety of malt and related products like beer.

Therefore, in addition to roasting the malt for practical applications, monitoring the a_w_, and controlling the environmental humidity and temperature, developing effective biological control and conservation measures in the malt industry chain according to the identified fungal communities can provide guidance for the safe use of malt.

## MATERIALS AND METHODS

### Sampling and fungal community enrichment.

Twenty batches of malt samples cultivated in Hebei province were collected from a market in China. The malt was divided into 10 batches of raw malt samples, labeled group M (M1 to M10), and 10 roasted malt samples, labeled group CM (CM1 to CM10). The raw and roasted samples from the same manufacturer were labeled with the same number, such as M1 and CM1. Detailed information is provided in Table 1. Samples from the same origin in Hebei province, China, were selected to eliminate the influence of other factors.

The 20 raw and roasted malt samples were homogenized to powder to prepare the fungal suspensions in parallel. Approximately 5.0 g of the sample powder was weighed into a 50-mL centrifuge tube, after which 25 mL phosphate-buffered saline (PBS) was added and mixed thoroughly by shaking for 2 h. The fungal communities attached to the malt surface were fully dispersed in the solution. After transient separation, the supernatant was transferred into a 10-mL centrifuge tube, followed by centrifugation at high speed for 10 min. The supernatant was discarded, and the fungal community precipitate was collected, transferred to a 2-mL centrifuge tube, and stored in a refrigerator at −80°C for subsequent analysis.

### Determination of the a_w_.

The a_w_ is a significantly important factor affecting fungal growth and fungal communities (46). Therefore, the a_w_ values of the 20 samples were determined using an HD-6 intelligent moisture activity measuring instrument (Wuxi Huake Instrument, Wuxi, China). The specific operational procedure was as follows: after stabilizing the detector, an appropriate number of raw and roasted malt samples were placed in petri dishes to determine the a_w_ value according to the operational guidelines.

### DNA extraction and SMRT sequencing.

Approximately 0.5 g of the enriched fungal community precipitate from each malt sample was obtained for total DNA extraction using the FastDNA spin kit for soil in accordance with the instructions of the manufacturer (MP Biomedicals, CA, USA). Agarose gel electrophoresis and an ND2000 spectrophotometer (Thermo Fisher Scientific, Waltham, MA, USA) were used to control the DNA yield and quality. The extracted DNA samples were stored at −80°C for further use.

The short reads of 250 to 400 bp of the ITS1 or ITS2 regions in the Illumina-based sequencing platform cause low species resolution and cannot fully identify the fungal community. Therefore, the third-generation SMRT sequencing technology was introduced for the molecular identification of the diverse fungal communities enriched in the malts. The ITS-based full-length amplicon covering the ITS1 and ITS2 variable regions was chosen as the amplification template, with ITS1F (5′-CTTGGTCATTTAGAGGAAGTAA-3′) and ITS4R (5′-TCCTCCGCTTATTGATATGC-3′) as the upstream and downstream primers, respectively (47). A 20-μL PCR mixture consisting of 4 μL of 5× FastPfu buffer, 2 μL of 2.5 mM deoxynucleoside triphosphates (dNTPs), 0.8 μL of 5 μM forward primer, 0.8 μL of 5 μM reverse primer, 0.4 μL of FastPfu DNA polymerase, 0.2 μL of bovine serum albumin (BSA), 10 ng of template DNA, and an appropriate volume of DNase-free water was applied. The specific PCR amplification procedure was as follows: (i) 3 min at 95°C; (ii) 35 cycles of denaturation at 95°C for 30 s, annealing at 55°C for 30 s, and extension at 72°C for 45 s; and (iii) 10 min at 72°C, followed by 10°C until halted by the user. After amplification, 2% agarose gel electrophoresis was employed to monitor the quality of the PCR product. Finally, the subsequent amplicons were sequenced on a PacBio-based SMRT sequencing platform (Pacific Biosciences, Menlo Park, CA, USA) from MajorBio Bio-pharm Technology Co. Ltd. (Shanghai, China).

### Sequence processing.

The circular consensus sequence (CCS) reads were generated via the raw PacBio sequencing data using SMRTLink 8.0. Then, the CCS reads were barcode identified and length filtered. Sequences outside a range of 300 bp to 900 bp were removed, and the optimized CCSs were retained for further downstream analyses. OTUs with a 97% similarity cutoff were clustered using UPARSE version 7.1 (43), while the chimeric sequences were identified and removed. The taxonomy of each representative OTU sequence was analyzed via RDP Classifier (version 2.11) against the UNITE database (release 8.0, https://unite.ut.ee/), using a confidence threshold of 0.7. All the analyses were conducted on the MajorBio Cloud platform (https://cloud.majorbio.com/).

### Detection of the mycotoxins.

To reveal the relationships between the identified fungi (especially toxigenic fungi) and the secondary metabolites, six of the mycotoxins most frequently monitored in food, namely, AFB_1_, AFB_2_, AFG_1_, AFG_2_, OTA, and ZEN, were analyzed in the 20 raw and roasted malt samples. All the samples were treated according to a QuEChERS (quick, easy, cheap, effective, rugged, safe) procedure to extract the mycotoxins. Then, the six target mycotoxins were detected based on a previously optimized UFLC-MS/MS method (48) using an AB Sciex Qtrap 5500 hybrid triple quadrupole/linear ion trap mass spectrometer (Sciex, Foster City, CA, USA) equipped with an electrospray ionization (ESI) source and a UFLC system (Shimadzu, Kyoto, Japan).

### Fungal communities and sequencing analysis.

The OTU annotation results were analyzed via pan- and core OTU curves to determine whether the sample size was large enough to evaluate the species diversity of the fungal communities. In addition, the *S*_obs_ index was used as an indicator to draw the rarefaction curves to evaluate the sequencing depth. The fungal community diversities in the malt samples were assessed based on the analysis of alpha diversity in terms of ACE, Shannon, Shannon evenness, and Good’s coverage parameters and the analysis of beta diversity by PCoA. The concrete composition of each taxon in each sample was represented by a fungal community distribution bar diagram. R tools were used to sketch the Venn diagram. ANOSIM was employed to test the statistical differences between the malt samples of the different (M and CM) groups. LEfSe was used to evaluate the specific species differences between the two groups (M and CM). RDA was employed to explore the correlations between the environmental factors and the fungi. A single-factor correlation network plot was created to examine the relationships among the identified fungal communities.

### qPCR validation of the detected fungal species.

The DNA of three samples (M4, CM6, and CM8) containing Candida tropicalis, Aspergillus proliferans, and Aspergillus sclerotiorum was extracted. About 30 mg of an enriched fungal suspension was collected from each sample for DNA extraction using a magnetic universal genomic DNA kit (Tiangen, China) in accordance with the standard protocol. The DNA quality and quantity were tested using a Nanodrop 2000 spectrophotometer (Thermo, USA) and 1% agarose gel electrophoresis.

All OTU sequences were aligned and assembled, with 97% as the minimum identity, using the CodonCode Aligner software. Species-specific regions in contigs were selected to design the primers. Then, three pairs of primers were designed according to the OTU sequences of Aspergillus proliferans, Aspergillus sclerotiorum, and C. tropicalis, using Primer Premier 6.0 software (Table S1), while the specificity was tested by using BLAST. All primers were synthesized by Sangon Biotech (Shanghai, China). qPCR was performed using TB Green premix *Ex Taq* (TaKaRa, Japan) on the CFX96 real-time system (Bio-Rad, USA). The reaction mixture contained 12.5 μL of 2× TB Green mix, 2 μL of the forward and reverse primers (2.5 μM), and 2 μL of DNA template. Sterilized water was added to reach a volume of 25 μL. The cycling parameters were set up as follows: 95°C for 30 s, 95°C for 5 s and 55°C for 60 s, and 72°C for 60 s for 40 cycles.

### Safety assessment of the malt samples.

To verify the identified fungal communities (especially toxigenic fungi) and further evaluate the impact of the roasting treatment, the 20 samples were treated for moldy culture under the same conditions. About 4.0 g of the raw or roasted malt samples was collected into dry, sterile petri dishes and sprayed with an appropriate amount of water on the surface. The petri dishes were covered and placed in a constant-temperature incubator at 28°C for culturing. The samples were photographed every 24 h until all samples exhibited obvious mold.

### Data availability.

The raw data of DNA sequences have been uploaded to the National Center for Biotechnology Information Sequence Read Archive database (BioProject PRJNA774475), and the accession numbers of the 20 samples are SAMN22566488 to SAMN22566507.
