# Supplementary material for: Single-Molecule Real-Time Sequencing to Explore the Mycobiome Diversity in Malt
Source: Microbiol Spectr. 2022 Sep 26;10(5):e00511-22. doi: 10.1128/spectrum.00511-22 (PMC9603040; doi:10.1128/spectrum.00511-22)
Supplement: Supplemental file 1 — Tables S1 and S2; Fig. S1 to S4. Download spectrum.00511-22-s0001.pdf, PDF file, 0.5 MB [file spectrum.00511-22-s0001.pdf]

Supplementary Materials

Table S1. Specific primers of 3 fungal species for the real-time quantitative PCR.

Name [a]	Sequences (5'→3')	Length/bp	Amplified length/bp
C. tro-F	TTTGAGCGTCATTTCTCC	18	192
C. tro-R	CCTGATGGGCGACTTGAA	18	
A. pro-F	CATTTCGCTGCGTTCTTC	18	122
A. pro-R	GTTTACAATCAGAAGCCG	18	
A. scl-F	ACAAGAGCGGGTGACAAA	18	238
A. scl-R	TAAAGCGACGCAAGAAGT	18	

[a] 3 fungal species: *Aspergillus proliferans*, *Aspergillus sclerotiorum*, and *Candida tropicalis*.

Table S2. Ct value of 3 fungal species for the real-time quantitative PCR.

Fungi	Ct	Average	RSD (%)
Candida tropicalis[1]	19.09		
Candida tropicalis[2]	19.30	19.02	1.40
Candida tropicalis[3]	18.66		
Aspergillus proliferans[1]	25.61		
Aspergillus proliferans[2]	25.72	25.66	0.17
Aspergillus proliferans[3]	25.66		
Aspergillus sclerotiorum[1]	27.81		
Aspergillus sclerotiorum[2]	27.46	27.61	0.53
Aspergillus sclerotiorum[3]	27.57		

1, 2, 3: three parallel experiments.

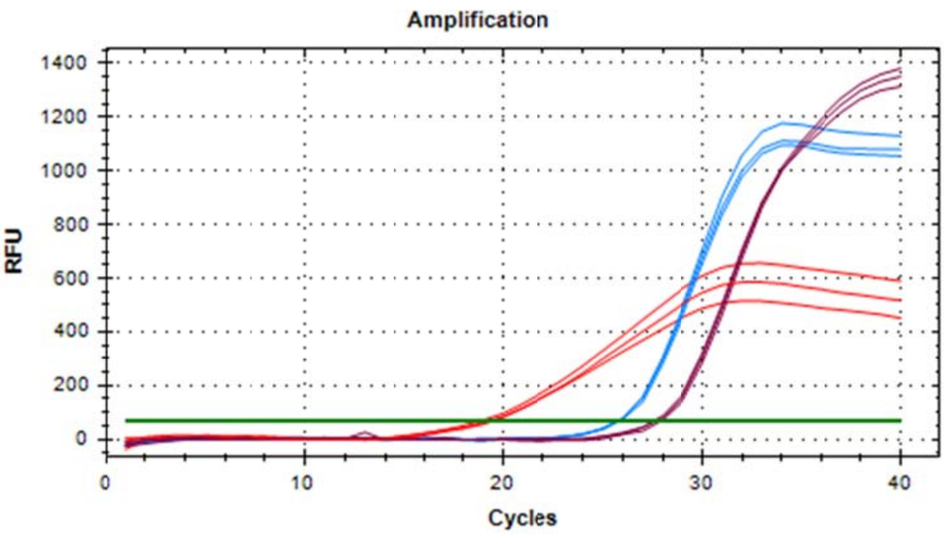

Figure S1. Amplification curves of three fungal species. The red curve, blue curve, and purple curve represent *Candida tropicalis*, *Aspergillus proliferans*, and *Aspergillus sclerotiorum*, respectively.

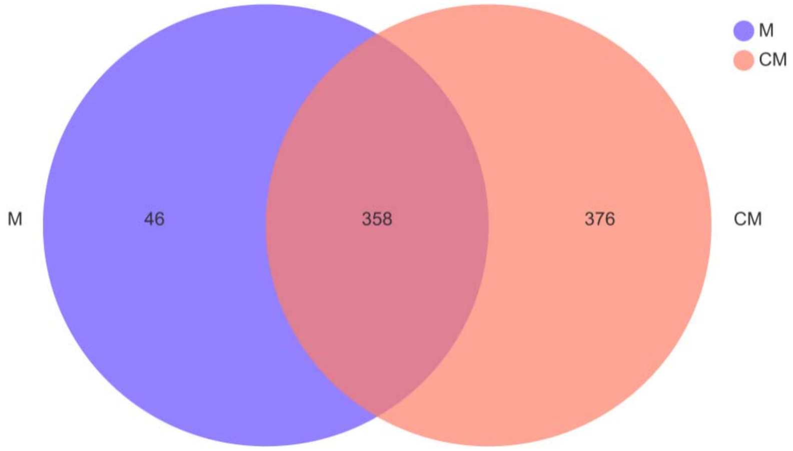

Figure S2. Venn graph of shared and exclusive OTUs in malts of groups M and CM.

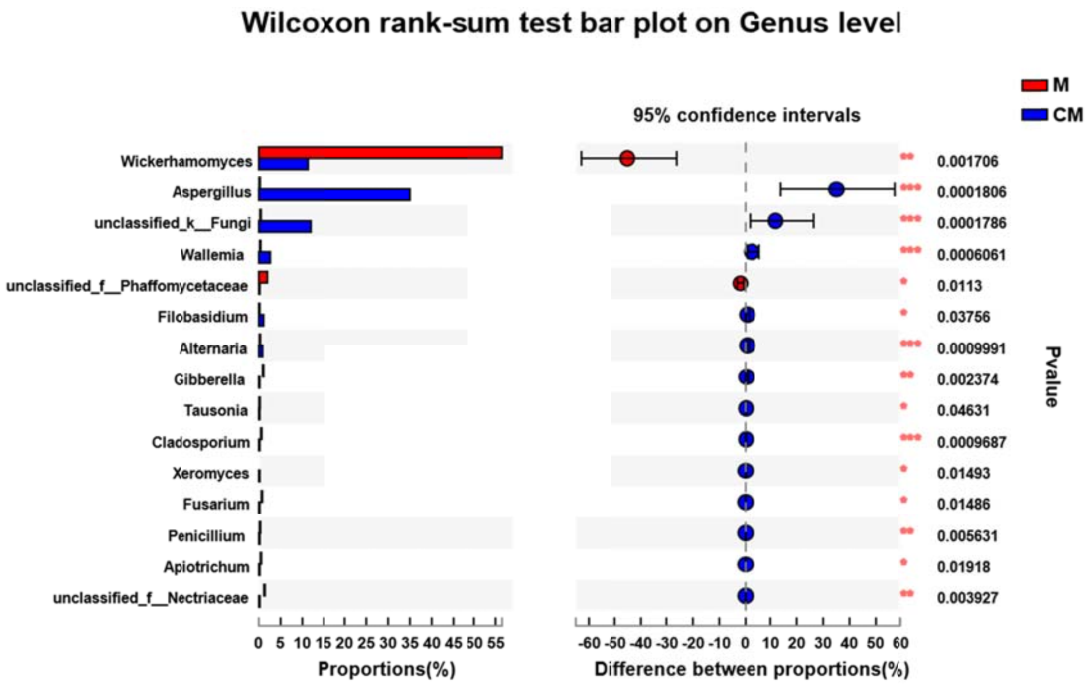

Figure S3. Wilcoxon rank-sum test bar plot presenting the fungal communities in raw and roasted malts. The top 15 genera with significant differences between the two groups (the M group and the CM group) were listed on the left with *P* values corresponding to the differential genus on the right.

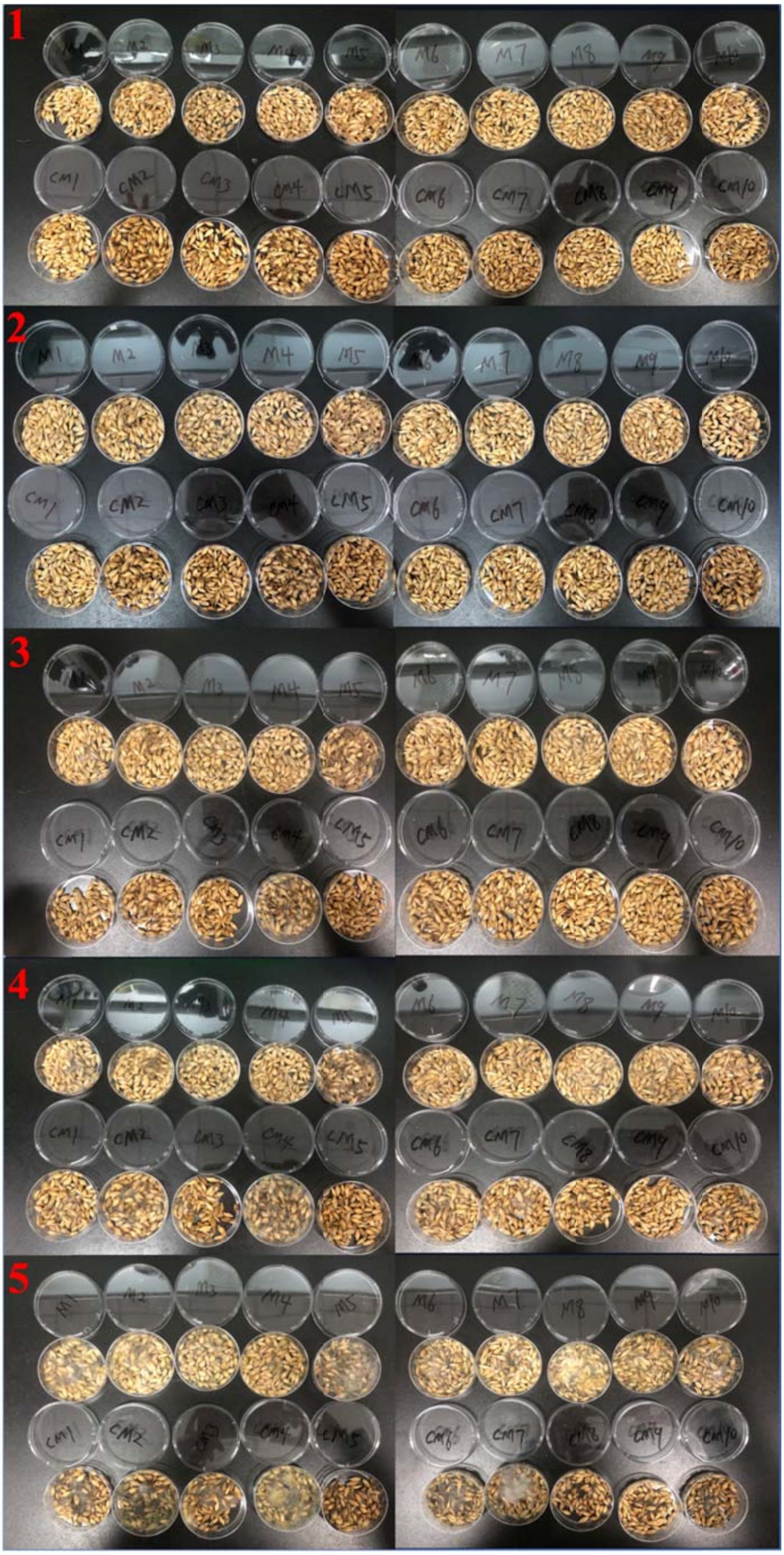

Figure S4. Mildewing observation of raw and roasted malt samples within 5 days.
